# Pernicious Anemia Associated Cobalamin Deficiency and Thrombotic Microangiopathy: Case Report and Review of the Literature

**DOI:** 10.1155/2017/9410727

**Published:** 2017-02-06

**Authors:** Farhanah Yousaf, Bruce Spinowitz, Chaim Charytan, Marilyn Galler

**Affiliations:** NewYork-Presbyterian/Queens, Flushing, NY, USA

## Abstract

A 43-year-old Hispanic male without significant previous medical history was brought to emergency department for syncope following a blood draw to investigate a 40 lbs weight loss during the past 6 months associated with decreased appetite and progressive fatigue. The patient also reported a 1-month history of jaundice. On examination, he was hemodynamically stable and afebrile with pallor and diffuse jaundice but without skin rash or palpable purpura. Normal sensations and power in all extremities were evident on neurological exam. Presence of hemolytic anemia, schistocytosis, thrombocytopenia, and elevated lactate dehydrogenase (LDH) was suggestive of thrombotic thrombocytopenic purpura (TTP). However, presence of leukopenia, macrocytes, and an inadequate reticulocyte response to the degree of anemia served as initial clues to an alternative diagnosis. Two and one units of packed red blood cells were transfused on day 1 and day 3, respectively. In addition, one unit of platelets was transfused on day 2. Daily therapeutic plasma exchange (TPE) was initiated and continued until ADAMTS-13 result ruled out TTP. A low cobalamin (vitamin B12) level was evident at initial laboratory work-up and subsequent testing revealed positive intrinsic factor-blocking antibodies supporting a diagnosis of pernicious anemia with severe cobalamin deficiency. Hematological improvement was observed following vitamin B12 supplementation. The patient was discharged and markedly improved on day 9 with outpatient follow-up for cobalamin supplementation.

## 1. Introduction

Microangiopathic hemolytic anemia (MAHA) refers to any hemolytic anemia associated with fragmented red blood cells (schistocytes) and small vessel pathology. Thrombotic microangiopathy (TMA) includes heterogeneous disorders characterized by MAHA, thrombocytopenia, and organ damage due to microvascular occlusion [1].

Thrombotic thrombocytopenic purpura (TTP) and hemolytic uremic syndrome (HUS) are two types of TMAs which may be fatal without prompt recognition and treatment. Thrombotic thrombocytopenic purpura (TTP), first described in 1924 [2], is a rapidly progressive and life-threatening condition which, in the past, was characterized by a classic pentad of MAHA, thrombocytopenia, fever, renal dysfunction, and neurologic abnormalities. The incidence of TTP in the United States is on the rise and is estimated to be about 4 cases per 1000000 [3]. It can be a familial or acquired idiopathic TTP and it may be an isolated episode or a recurring event. Chronic relapsing type-TTP is most often seen in infants and children, while two-thirds of patients with idiopathic single-episode TTP are adults [4–6].

TTP results from immunoglobulin G (IgG) and autoantibodies to von Willebrand factor-cleaving protease (ADAMTS-13). ADAMTS-13 is required for degradation of the highly adhesive and reactive forms of large von Willebrand factor multimers which can be up to approximately 20000 kDa in size [7, 8]. The current standard of care in the management of acquired TTP is daily therapeutic plasma exchange (TPE) with fresh frozen plasma (FFP) replacement until the resolution of neurological symptoms (if applicable), return of platelet count greater than 150 k/uL, and LDH near normal range for 2 to 3 consecutive days [9]. TPE works by removing ADAMTS-13 antibodies and cytokines and infusing FFP containing functional ADAMTS-13 [10]. Its role is unclear and some reports suggest that it may be harmful in the management of infection associated HUS which results from toxin-mediated endothelial injury [9]. On the other hand, noninfectious HUS, which results from excessive alternative complement activation, may benefit from plasma infusion or TPE, while Eculizumab is the preferred therapeutic strategy for controlling atypical HUS and limiting renal damage [11, 12].

TTP and other hemolytic microangiopathies are often severe with significant morbidity and mortality rates remaining as high as 10–20% in spite of TPE [13, 14]. The hematological features of TTP, such as thrombocytopenia, hemolytic anemia, and schistocytosis, suggest a wide differential diagnosis, including, but not limited to, disseminated intravascular coagulation, HUS, and autoimmune hemolysis. The diagnosis of TTP may be challenging since certain conditions can mimic the signs and symptoms of TTP [15].

TMA has been reported in association with pregnancy [16–19], human immunodeficiency virus (HIV) [20], malignancy and chemotherapeutic agents [21, 22], malignant hypertension [23], systemic lupus erythematosus (SLE) [24], bone marrow transplantation [25, 26], and drugs [27] including antiplatelets [28, 29], antimalarials [30], and immunosuppressants [31, 32].

Cobalamin (vitamin B12) deficiency induced TMA is a rare condition which closely resembles the clinical features of TTP such as thrombocytopenia, hemolytic anemia, and schistocytosis. This report presents a case of cobalamin deficiency induced TMA in a patient initially suspected of having TTP, with important implications for the work-up and treatment of patients with suspected TTP.

## 2. Case Description

A 43-year-old Hispanic male with no past significant medical history presented to the emergency department (ED) with syncope, progressive fatigue, decreased appetite, weight loss of 40 lbs over the past six months, and one-month history of jaundice. The patient denied any chest pain, dyspnea, fever, cough, tingling, numbness, headache, visual changes, focal weakness, abdominal pain, bleeding, leg swelling, rash, changes in urinary or bowel habits, or sick contacts or any history of anemia. Upon initial physical examination, the patient was afebrile with a blood pressure of 135/69 mmHg and tachycardia. The patient had pallor and diffuse jaundice without hepatosplenomegaly, skin rash, or palpable purpura. On neurological exam, the patient was alert and oriented to person, place, and time with normal sensation and muscle strength in all extremities. Examination of the remaining systems was unremarkable.

Initial laboratory (Table 1) investigations revealed anemia, reticulocytosis, increased mean corpuscular volume of the red blood cells, thrombocytopenia, leukopenia, and a markedly elevated lactate dehydrogenase (LDH) level. Schistocytes, anisocytes, macrocytes, microcytes, ovalocytes, helmet, and tear drop cells were present on the peripheral blood smear. Bilirubin, aspartate aminotransferase (AST), and D-dimers were elevated, while haptoglobin was decreased. Iron indices were consistent with hemolytic anemia. Renal function was preserved. Hepatitis panel was positive for hepatitis B surface antigen and core antibody, confirming a diagnosis of active hepatitis B infection. An abdominal ultrasound revealed cholelithiasis, splenomegaly, hepatic steatosis, and a nonobstructive renal calculus. The presence of hemolytic anemia, schistocytosis, thrombocytopenia, and elevated LDH in this patient were suggestive of TTP or atypical HUS, possibly related to hepatitis B.

On days 1 and 3 of admission, the patient was transfused with two and one units of packed red blood cells, respectively. On day 2, the patient was transfused with one unit of platelets. The combination of a peripheral blood smear suggestive of MAHA and thrombocytopenia serves as an acceptable indication for the initiation of TPE [9, 33] and, therefore, the patient underwent two sessions of TPE with fresh frozen plasma replacement on days 2 and 3. The platelet patterns following TPE in TTP/HUS can vary [34] and therefore alternative etiologies were dismissed until TTP could be ruled out. Steroids and antihistamines were added to the TPE regimen because the patient developed a skin reaction presumed to be secondary to the blood products.

Cobalamin deficiency was noted at admission laboratory evaluation but was not accepted as the underlying cause of patient's clinical condition. However, one dose of 1000 mcg of cyanocobalamin was administered intramuscularly on day 3. Additional TPE sessions were continued until day 5, while ADAMTS-13 report was pending. The platelet count remained in a range of 18–37 K/uL despite daily TPE. On day 6, a normal ADAMTS-13 report was reported and TTP was ruled out. Platelet count improved to 90 and 138 K/uL on days 8 and 9, respectively.

Further investigations were also positive for intrinsic factor-blocking antibodies and an elevated gastrin level consistent with a diagnosis of pernicious anemia. The patient's condition improved markedly and the patient was discharged on day 9 with outpatient follow-up for vitamin B12 supplementation.

## 3. Discussion

Cobalamin deficiency induced TMA poses a diagnostic hurdle for clinicians encountering patients presenting with thrombocytopenia, hemolytic anemia, and schistocytosis. Although the differential diagnosis should always seek ruling out the most life-threatening conditions first, measurement of cobalamin and methylmalonic acid level to the current diagnostic panel for the evaluation of TTP can direct physicians to proper diagnosis and management [9]. Moreover, our case report emphasizes the limited awareness among physicians regarding cobalamin deficiency induced TMA, since TPE was continued in spite of the evidence of severe cobalamin deficiency. A lack of awareness of this entity is also illustrated by the absence of a recommendation for cobalamin measurement in suspected TTP in the 2012 British Journal of Hematology Guidelines [35]. TTP carries a mortality rate of 90% without TPE [35] and a delay in the initiation of TPE serves as an independent risk factor for short-term mortality [36]. However, a mortality rate of 2.3% and a major complications rate of 24% have been attributed to TPE [37]. Our patient had evidence of active hepatitis B infection with mild liver dysfunction which complicated the diagnosis because hepatitis can be associated with thrombocytopenia and aplastic anemia [38, 39]. Moreover, TMA has been reported in association with hepatitis C related heat insoluble cryoglobulinemia [40]. Therefore, the continuation of TPE until TTP could be excluded was considered to outweigh potential risks in our patient. The exclusion of TTP in the presence of cobalamin deficiency may be further complicated by a pre-TPE low ADAMTS-13 activity level [41, 42].

Cobalamin is vital for DNA synthesis and hematopoietic cell division. Its ingestion occurs in the protein-bound state and, thus, requires release by gastric acid and pancreatic protease. Cobalamin is most efficiently absorbed in the distal ileum bound to intrinsic factor, a protein produced by the gastric parietal cells. Passive diffusion alone contributes to absorption of only 1–5% of the daily intake of cobalamin [43]. Cobalamin circulates in the plasma bound to either holotranscobalamin (6–20%) or holohaptocorrin (80%). Holotranscobalamin delivers cobalamin to all DNA-synthesizing cells and serves as the earliest marker of cobalamin deficiency [44].

Cobalamin derivatives serve as cofactors in two major cellular reactions. The generation of methionine from homocysteine requires cytoplasmic methylcobalamin and the conversion of methyl-malonyl-coenzyme A to succinyl-coenzyme A requires mitochondrial 5-deoxyadenosylcobalamin [45]. Hence, cobalamin depletion often leads to the accumulation of homocysteine and methylmalonic acid, which serve as surrogate markers of cobalamin deficiency. There is a lack of consensus regarding the gold standard assay for the determination of cobalamin levels [46]. Patients with pernicious anemia may exhibit falsely elevated serum cobalamin levels due to intrinsic factor antibody interference [47–49]. Homocysteine levels demonstrate poorer specificity than methylmalonic acid levels but increased levels of methylmalonic acid may not correlate with* clinical* cobalamin deficiency as was seen in a Danish study which reported a lack of significant difference in the prevalence of cobalamin deficiency related symptoms in individuals with methylmalonic acid ≥0.4 *μ*mol/L versus <0.4 *μ*mol/L [50]. In renal insufficiency, a normal to high cobalamin level cannot rule out* functional* deficiency [51, 52]. The evaluation of methylmalonic acid and holotranscobalamin levels can distinguish between functional cobalamin deficiency and cobalamin storage depletion but both of these markers may be falsely elevated in renal impairment [53–55]. Therefore, the interpretation of the markers of cobalamin deficiency warrants an assessment of renal function. In addition, holotranscobalamin, methylmalonic acid, and homocysteine levels are poorer predictors of the hematological response to vitamin B12 therapy compared to a low total cobalamin level [56]. Moreover, a total cobalamin level of 156–450 pmol/L cannot rule out cobalamin deficiency [57]. The presence of megaloblastic anemia is a nonspecific and insensitive reflection of vitamin B12 status [58, 59].

Cobalamin deficiency was traditionally thought to be a disease of elderly Caucasians but is now known to be common in multiple ethnic groups and ages [60]. The causes of cobalamin deficiency include pernicious anemia, dietary deficiency associated with vegetarian and vegan diets, postsurgical malabsorption, and malabsorption secondary to gastrointestinal pathology [61]. The daily requirement of cobalamin is 6–9 micrograms. Because the body stores between 2 and 5 milligrams of cobalamin, its deficiency usually develops slowly and manifests after multiple years of inadequate dietary intake or malabsorption [62].

The prevalence of cobalamin deficiency is 3–40% depending on the definition and biochemical markers used as well as the population explored [63–66]. Pernicious anemia is a common cause of cobalamin deficiency and results from autoimmune destruction of parietal cells and intrinsic factor deficiency related cobalamin malabsorption. The development of hypochlorhydria due to the loss of parietal cells leads to an increase in gastrin levels. The prevalence of pernicious anemia is 0.1% in the general population which increases to 1.9% in patients aged older than 60 years [67, 68] and it contributes to 20–50% of cobalamin deficiency cases [69]. Both parenteral and oral doses of cyanocobalamin, hydroxocobalamin, or methylcobalamin may be used in pernicious anemia related cobalamin deficiency [70, 71], although hydroxocobalamin administration is associated with better uptake and storage compared to other forms [72]. Oral cobalamin doses of 1000 mcg per day lead to more than adequate absorption via passive diffusion to meet daily requirements in intrinsic factor deficient patients [43]. Nonetheless, parenteral cobalamin replacement is preferred in patients with cobalamin deficiency related neurological deficits. The typical vitamin B12 replacement approach usually consists of a monthly cobalamin dose of 100–1000 mcg [72].

TMA develops in about 2.5% of cobalamin deficiency cases [73, 74]. The pathogenesis of cobalamin deficiency induced TMA is poorly understood but may involve homocysteine and/or its derivatives initiating endothelial injury and dysfunction [75]. Mild-to-moderate hyperhomocysteinemia has been shown to trigger the activation of coagulation cascade, alter endothelial adhesive properties, and impair the vascular response to L-arginine [76]. The activation of the coagulation pathway may increase D-dimer levels as was seen in our patient as well as in another case report in which D-dimer levels correlated with the degree of schistocytosis [77]. In addition, macrocytic erythrocytes resulting from cobalamin deficiency have reduced deformability which predisposes to entrapment in the microcirculation [78]. Furthermore, ineffective erythropoiesis secondary to cobalamin deficiency leads to intramedullary hemolysis, indirect hyperbilirubinemia, elevated LDH, low haptoglobin, and microangiopathic features on peripheral blood smear. Taken together, these events consequently result in fragmentation of erythrocytes and manifest as TMA. Immune dysfunction is suspected to be involved in the development of TMA, since the majority of cobalamin deficiency induced TMA cases have been reported in patients with pernicious anemia [74, 77, 79–88]. However, a key role of immune dysfunction in cobalamin deficiency induced TMA seems less likely because patients with autosomal recessive disorders of cobalamin activation [89, 90] and food-cobalamin malabsorption have also presented with TMA [41, 88].

Noël et al. compared clinical and biochemical parameters in seven patients with cobalamin deficiency induced TMA and seven patients with TTP [88]. First, none of the seven patients with cobalamin deficiency induced TMA exhibited acute renal failure, while all of the patients with TTP did. Second, none of the seven patients with cobalamin deficiency induced TMA presented with severe neurological symptoms, such as drowsiness or coma, as is common in TTP. Third, LDH levels were much higher and bilirubin levels were much lower than expected in the cobalamin deficiency induced TMA group compared to the TTP group. Fourth, the TTP group exhibited an elevated reticulocyte count, which was absent in the cobalamin deficiency induced TMA group.

TTP is associated with significant mortality and demands early diagnosis and prompt initiation of TPE. The diagnosis of TTP can be supported by a deficient ADAMTS-13 activity level but the long turnaround times of ADAMTS-13 testing limit its utility and TTP largely remains a clinical diagnosis. Although fluorescence resonance energy transfer (FRETS) method for determining ADAMTS-13 activity is a rapid technique, it is not widely available. Alternative underlying causes, especially cobalamin deficiency, should be explored at presentation in all cases of suspected TTP. The 2012 British Journal of Hematology Guidelines recommends that serological testing for HIV, hepatitis B and hepatitis C, autoantibody screen, and pregnancy test, when appropriate, be performed at presentation for patients with suspected TTP, given the reported correlation between HIV infection, viral hepatitis, and thrombocytopenia [35]. Given the emergence of multiple case reports of cobalamin deficiency induced TMA, we strongly recommend the addition of cobalamin and methylmalonic acid testing to the diagnostic panel. Aggressive interventions [86, 91] such as TPE, Rituximab, and steroids may be avoided by increasing physician awareness regarding cobalamin deficiency induced TMA and routine screening for cobalamin deficiency. Furthermore, empirical oral cobalamin supplementation in TTP suspected cases may be considered in light of the limited specificity of screening for cobalamin deficiency and the lack of evidence of cobalamin toxicity.

## 4. Conclusion

Cobalamin deficiency induced TMA is a rare condition but should be considered in all patients presenting with clinical and laboratory features of TTP. The presence of leukopenia, macrocytes, and a slightly elevated reticulocyte count are initial clues that raise suspicion for cobalamin deficiency induced TMA. The emergence of multiple case reports of cobalamin deficiency induced TMA warrants the addition of cobalamin and methylmalonic acid testing in all cases of suspected TTP. By adding cobalamin and methylmalonic acid testing to all TMA screens, the diagnostic delays in detecting and treating cobalamin deficiency as well as the potential TPE related iatrogenic harm may be prevented.

## Figures and Tables

**Table 1:** Laboratory investigations.

Investigation	At admission	During hospitalization	At discharge	Reference range
*Hematology studies*				

Hemoglobin (g/dL)	5.0		9.3	13.5–17.5
Reticulocyte count (%)	2.6			0.2–2.0
Mean corpuscular volume (fL)	111		107	80–100
Platelets (K/uL)	24		138	150–400
White cell count (K/uL)	2.77		7.96	4.8–10.8
vWF protease activity/ADAMTS-13 (%)	88			68–163
BUN (mg/dL)	21			6–20
Creatinine (mg/dL)	0.69			0.7–1.2
BUN/creatinine ratio	30			10–20
GFR	125			

*Coagulation studies*				

Prothrombin time	12.4			
APTT	24.2			
D-Dimer (ng/mL)	668			<209

*Chemistry studies*				

LDH (U/L)	3906			135–225
Bilirubin [indirect] (mg/dL)	4.1			0–0.9
AST (U/L)	69			5–40
ALT (U/L)	40			5–41
Alkaline phosphatase (U/L)	65			40–130
Cyanocobalamin (pg/mL)	38			211–946
Folate (ng/mL)	14			7.3–20
Gastrin (pg/mL)			1077	≤100
Methylmalonic acid (nmol/L)			1491	87–318
Haptoglobin (mg/dL)	<10			30–200

*Flow cytometry*				

PNH w/FLAER		Negative		

*Serology studies*				

Intrinsic factor blocking Ab		Positive		Negative
Hepatitis B surface antigen	Positive			Negative
HBV core Ab	Reactive			Nonreactive
HIV	Nonreactive			Nonreactive
Parvovirus B19 (IgG/IgM)	Negative			Negative
EBV			Negative	
CMV			Negative	
Tissue transglutaminase Ab (IgA/IgG)			Negative	

*Urine studies*				

Urinalysis		Unremarkable	Unremarkable	
Urine culture			No growth	

*Other*				

Stool guaiac	Negative			
Peripheral blood smear^*∗*^	Schistocytes, anisocytes, macrocytes, microcytes, ovalocytes, helmet, and tear drop cells			
Abdominal ultrasound^*∗*^	Cholelithiasis, splenomegaly, hepatic steatosis, and nonobstructive renal calculus			

^*∗*^Peripheral blood smear and abdominal ultrasound findings at admission; LDH: lactate dehydrogenase; EBV: Epstein-Barr virus; CMV: cytomegalovirus; HIV: human immunodeficiency virus; HBV: hepatitis B virus; PNH: paroxysmal nocturnal hemoglobinuria [antibodies were directed against CD33, CD45, and glycophorin A (for gating) and CD14, CD16, CD24, CD55, and CD59 as well as FLAER for flow cytometric assessment of glycophosphatidylinositol- (GPI-) linked molecules].
